# Axonal Signaling Proteins Take the ER Highway

**DOI:** 10.1371/journal.pbio.1000504

**Published:** 2010-10-05

**Authors:** Richard Robinson

**Affiliations:** Freelance Science Writer, Sherborn, Massachusetts, United States of America

**Figure:**
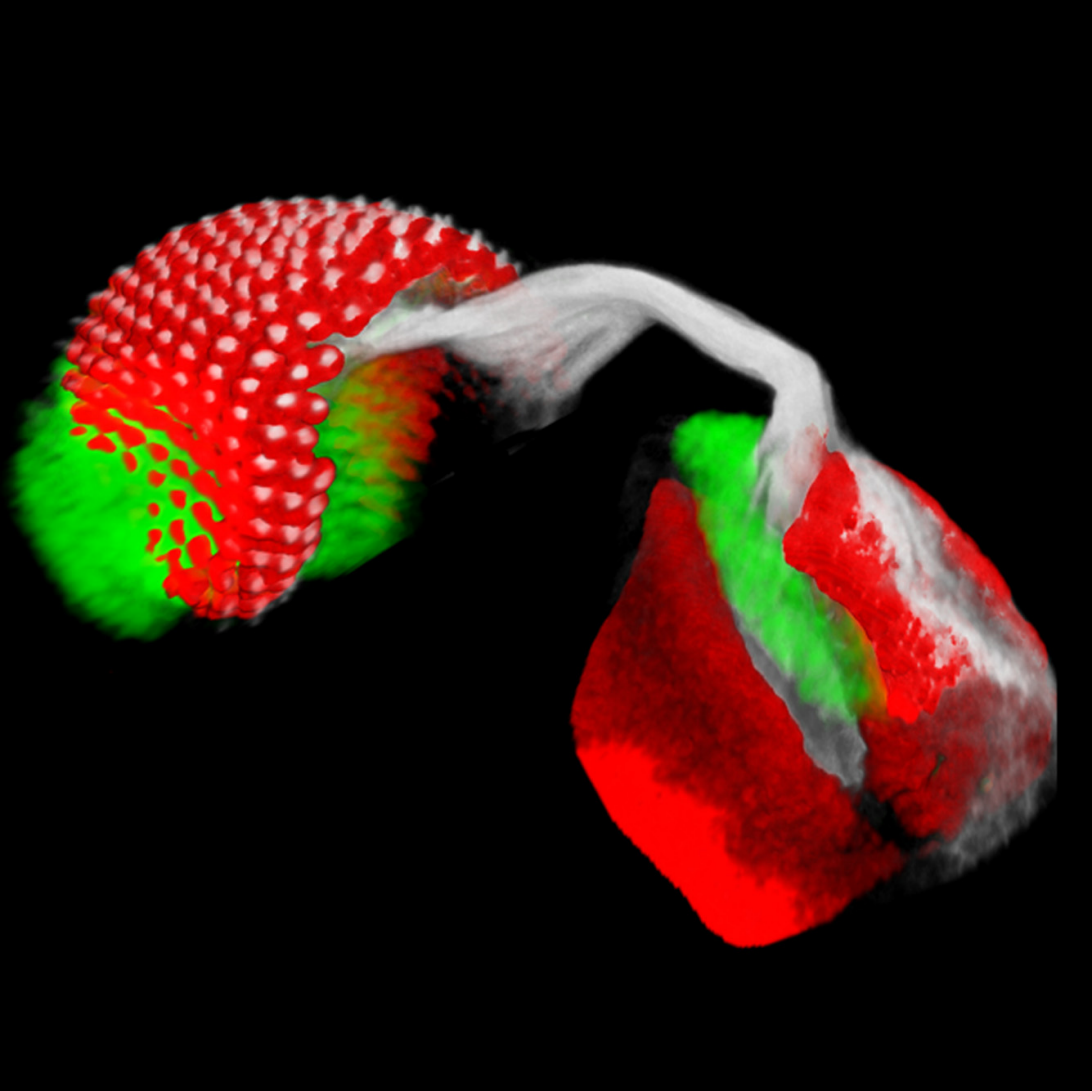
Localization of the *Drosophila* EGF receptor ligand and its processing machinery within the endoplasmic reticulum of photoreceptor cells is essential for axonal trafficking and secretion of the ligand at the outer layer of the brain. Neural cell fates are marked in red, axon fascicles in gray.

It's a long way from the cell body of a neuron to its axonal tip—several feet or more in the longest human neurons, and, even in the smallest ones, the length of the axon can be hundreds of times the width of the cell. Transporting cargo along that distance is primarily the job of microtubules of the cytoskeleton, but it is aided in some small measure by the endoplasmic reticulum (ER), a system of internal membranes that is continuous with the nuclear membrane and pervades the cell. But the ER has not been thought to play much of a role in transport of proteins over such long distances. That view has begun to change recently, however, as a small number of proteins have been shown to be trafficked within the ER along the length of the axon. In this issue of *PLoS Biology*, Shaul Yogev, Eyal Schejter, and Ben-Zion Shilo add to that list and show that it includes a cluster of proteins whose combined action plays a key role in development of the fly visual system.

In the developing visual system of Drosophila, the axons of photoreceptor neurons grow until they reach a target layer on the outer surface of the brain called the lamina. There, they release a series of proteins that trigger neurogenesis in lamina precursor cells. One of these factors is a ligand for the epidermal growth factor receptor. The ligand, called Spitz, requires an internal chaperone called Star to get it where it is going and must be cleaved by a protease called Rhomboid-3 before it is released in its active form. The authors began their investigation by showing that there was no Rho-3 messenger RNA in the axon, suggesting that, unlike many other proteins at the axon terminus, Rho-3 is not synthesized from mRNA transported along microtubules to the axon tip.

Within the cell body, the region that surrounds the photoreceptor cell's nucleus, Rho-3 is known to be linked to the ER, while another Rho, called Rho-1, is not. While Rho-1 can cleave Spitz, it does so only at the cell body, not at the axon terminus. What accounts for that specificity? The authors identified membrane targeting sequences on each Rho; when they switched them, the two proteins traded places. Rho-3 that could not bind to the ER could not process Spitz at the axon terminus, while Rho-1 with an ER-targeting signal could, indicating that ER localization of Rho-3 is the critical event in shuttling it to the end of the axon where it can cleave Spitz. By tagging Rho-3 with a fluorescent protein and an ER-specific marker, they showed that the protein was continuously distributed along the length of the axon, confirming the importance of the ER in trafficking of Rho-3 to the end of the axon. Furthermore, Rho-3 associated in the ER with both Spitz and its chaperone Star within the axon. Once at the terminus, Rho-3 and Spitz exited the ER and entered a secretory vesicle, where Rho-3 cleaved Spitz, allowing it to be secreted and to drive lamina neurogenesis.

One potential advantage of ER-facilitated trafficking, the authors suggest, may be that it ensures greater control over the transport and release of the signal, preventing release either in the wrong place or at the wrong time in development. The results of the current study indicate that the axonal endoplasmic reticulum functions as a conduit for at least one important group of signaling proteins in at least one important developmental process. More likely, there are other ER-trafficked proteins important in other processes awaiting discovery.

**Yogev S, Schejter ED, Shilo B-Z (2010) Polarized Secretion of** ***Drosophila*** **EGFR Ligand from Photoreceptor Neurons Is Controlled by ER Localization of the Ligand-Processing Machinery. doi:10.1371/journal.pbio.1000505**

